# Carbon-infiltrated carbon nanotubes inhibit the development of *Staphylococcus aureus* biofilms

**DOI:** 10.1038/s41598-023-46748-y

**Published:** 2023-11-08

**Authors:** Lucy C. Bowden, Jocelyn G. W. Evans, Katelyn M. Miller, Anton E. Bowden, Brian D. Jensen, Sandra Hope, Bradford K. Berges

**Affiliations:** 1 Department of Microbiology and Molecular Biology, Brigham Young University, Provo, UT 84602 USA; 2 Department of Statistics, Brigham Young University, Provo, UT 84602 USA; 3 Department of Mechanical Engineering, Brigham Young University, Provo, UT 84602 USA

**Keywords:** Materials science, Materials for devices, Nanoscale materials, Microbiology, Antimicrobials, Bacteria, Biofilms

## Abstract

*Staphylococcus aureus* forms biofilms that cause considerable morbidity and mortality in patients who receive implanted devices such as prosthetics or fixator pins. An ideal surface for such medical devices would inhibit biofilm growth. Recently, it was reported that surface modification of stainless steel materials with carbon-infiltrated carbon nanotubes (CICNT) inhibits the growth of *S. aureus* biofilms. The purpose of this study was to investigate this antimicrobial effect on titanium materials with CICNT coated surfaces in a variety of surface morphologies and across a broader spectrum of *S. aureus* isolates. Study samples of CICNT-coated titanium, and control samples of bare titanium, a common implant material, were exposed to *S. aureus.* Viable bacteria were removed from adhered biofilms and quantified as colony forming units. Scanning electron microscopy was used to qualitatively analyze biofilms both before and after removal of cells. The CICNT surface was found to have significantly fewer adherent bacteria than bare titanium control surfaces, both via colony forming unit and microscopic analyses. This effect was most pronounced on CICNT surfaces with an average nanotube diameter of 150 nm, showing a 2.5-fold reduction in adherent bacteria. Since *S. aureus* forms different biofilm structures by isolate and by growth conditions, we tested 7 total isolates and found a significant reduction in the biofilm load in six out of seven *S. aureus* isolates tested. To examine whether the anti-biofilm effect was due to the structure of the nanotubes, we generated an unstructured carbon surface. Significantly more bacteria adhered to a nonstructured carbon surface than to the 150 nm CICNT surface, suggesting that the topography of the nanotube structure itself has anti-biofilm properties. The CICNT surface possesses anti-biofilm properties that result in fewer adherent *S. aureus* bacteria. These anti-biofilm properties are consistent across multiple isolates of *S. aureus* and are affected by nanotube diameter. The experiments performed in this study suggest that this effect is due to the nanostructure of the CICNT surface.

## Introduction

*Staphylococcus aureus* is a common gram-positive bacterium that leads to nearly 20,000 deaths each year in the US^1^. *S. aureus* forms biofilms, which are surface-associated assemblages of bacteria embedded in an extracellular matrix. Infections with established biofilms are very difficult to treat with traditional antibiotic regimens due to limited diffusion of antibacterial agents through the biofilm matrix. *S. aureus* biofilms cause severe infections in healthcare settings and are particularly problematic in the context of implanted hardware such as fixator pins or artificial joints.

Orthopedic implants and prostheses are becoming more and more common—in 2018 over 1 million hip and knee implants were performed in the US alone^2^. Although these implants can relieve pain and restore freedom of movement, they are susceptible to post-operative periprosthetic joint infection (PJI), which is responsible for about 30% of implant failure^3^. Up to 70% of PJI cases are caused by *S. aureus*^4–7^*.* Infection rates are even worse for external fixator pins, where up to 80% of all patients experience a pin tract infection and most of these are caused by *S. aureus*^8,9^. Current efforts to reduce infection rates have proven insufficient as the yearly infection burden continues to rise^3^.

Titanium (Ti) is often used in medical hardware for its high mechanical strength, but this material also offers a ready surface for bacterial colonization and biofilm formation. An ideal implant surface would exhibit innate physical resistance to the adherence and formation of bacterial biofilms. This physical resistance to biofilm formation is due to the texture of the surface at the nanoscale. Structural biofilm resistance is an attractive alternative to antibiotics for biofilm control because it could reduce the need for antibiotic use, which would help reduce the development of antibiotic-resistant bacteria. Many natural surfaces possess structural antimicrobial properties, including dragonfly^10^ and cicada wings^11^, shark skin^12^, and lotus leaves^13,14^. Several synthetic analogs have been developed in an attempt to replicate the anti-biofilm effects of naturally biofilm-resistant surfaces^15–17^. While these materials have promising applications, they also suffer from limitations such as cytotoxicity to human cells and manufacturing difficulties, constraining their use in medical tools and implants. The search for a material that can overcome these limitations is ongoing, but one promising material is carbon-infiltrated carbon nanotubes (CICNT). The CICNT surface aims to replicate the structural biofilm resistance of naturally occurring antimicrobial surfaces.

Carbon Nanotubes (CNT) are nanostructured cylindrical lattices of hybridized carbon atoms. CNT possess impressive structural and mechanical properties that make them of interest in a variety of applications in biotechnology^18,19^. They are synthesized using chemical vapor deposition techniques, flowing ethylene gas at high temperatures over a substrate such as silicon, Ti, or stainless steel^20^.

Post-processing alteration of CNT is common for biological applications. When a post-processing carbon infiltration step is added, amorphous carbon is deposited on the nanotubes, resulting in carbon-infiltrated carbon nanotubes (CICNT). This infiltration step massively increases the diameter of the nanotube, resulting in a final volume ratio of around 99% bulk carbon to 1% CNT^21^, as well as providing substantial structural reinforcement that changes the mechanical behavior of the CNT forest from individual tubes to a cohesive structural layer.

The purpose of this study was to demonstrate that the CICNT surface has antimicrobial properties and to better understand these properties by determining whether changing nanotube size or the material at the nanotube/bacteria interface would affect bacterial adhesion. This was accomplished through the quantification of adhered *S. aureus* cells on CICNT as well as bare Ti by colony forming unit analysis. The CICNT surface offers a potential solution to the increasing burden of implant-associated infection.

## Results

### Optimizing CICNT diameter to reduce biofilm growth

**Figure 1:**
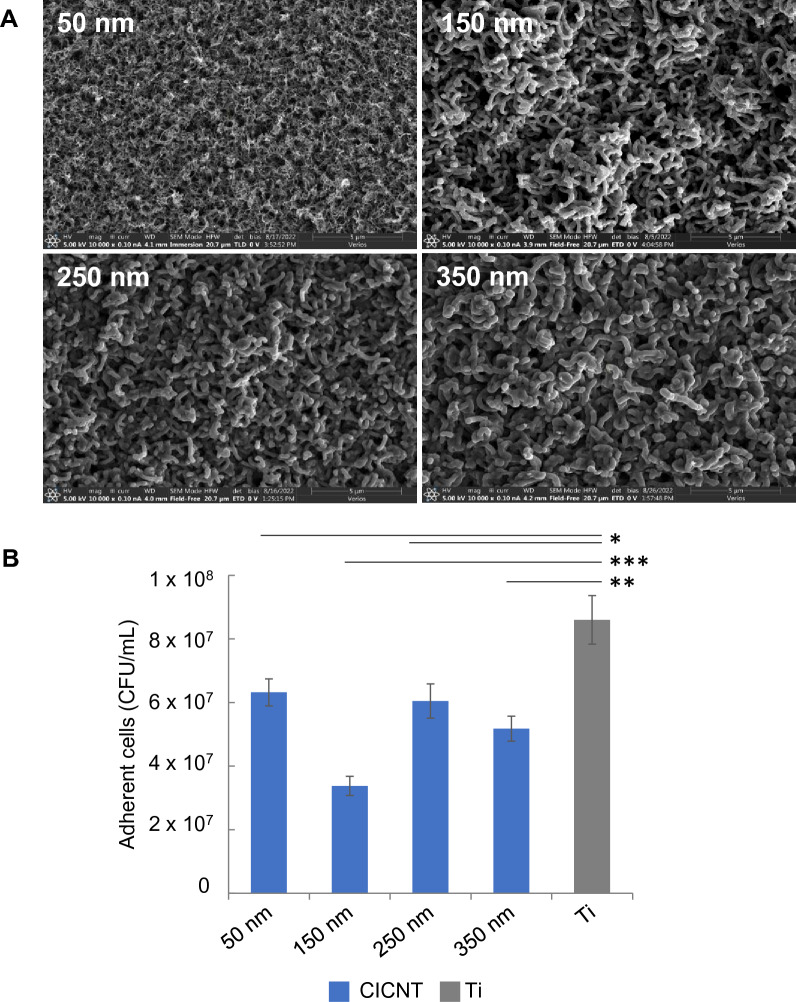
(**A**) Scanning electron micrographs of CICNT of various diameters at 10,000× magnification. (**B**) Colony forming units (CFU)/mL of bacteria (strain JE2) for CICNT of various diameters and bare titanium (Ti) + /− standard error. Note that CICNT is denoted in blue and bare Ti in grey in this and all subsequent figures. Bars represent n = 7 total samples and three independent experiments for each group. *p < 0.05, **p < 0.005, ***p < 0.0005.

We first investigated the optimal size parameters for CICNT biofilm resistance. The size of individual nanotubes can be manipulated by altering the amount of carbon infiltration. CICNT 9 × 9 mm squares (herein referred to as samples) of various diameters (50, 150, 250, and 350 nm, Fig. 1A) were prepared in order to quantify the effects of CICNT diameter on surface biofilm growth. *S. aureus* strain JE2 was grown on each surface for 36 h. JE2 was chosen because it is a USA300 strain, which is clinically relevant, and because it was found to have a biofilm matrix structure that was representative of many *S. aureus* isolates in quantities of polysaccharide, protein, and extracellular DNA^22^. After 36 h, the biofilm was washed to remove unadhered cells. Adherent cells were then removed from the sample surface and quantified by performing serial dilutions, then plating onto LB agar to count colony-forming units (CFU) of bacteria. Each diameter size of CICNT showed a significantly reduced number of bacteria as compared to a bare Ti control, ranging from a 1.4-fold reduction with 50 nm CICNT (*p* = 0.03) to a 2.5-fold reduction with 150 nm CICNT (p = 0.0005) after 36 h (Fig. 1B, Table 1). After determining that the 150 nm diameter was most effective, we used that size of CICNT for all future experiments.

**Table 1:** *P*-values from statistical comparison of results from CICNT of various diameters.

nm	50 nm	150 nm	250 nm	350 nm
50	–	0.0011	0.33	0.031
150		–	0.0032	0.0048
250			–	0.14
350				–

In an effort to better understand the mechanism behind the differential effect of nanotube diameter on *S. aureus* adhesion, we analyzed the wettability of each type of surface by measuring the water contact angle on samples of each nanotube size as well as bare titanium. We found that there was not a significant difference in water contact angle between nanotube size groups or bare Ti (Table 2, *p* = 0.46), indicating that wettability was not the causative factor behind the difference in bacterial adhesion.

**Table 2:** Average water contact angle +/− standard error.

	Water contact angle
50 nm	62.9 + /− 3.8
150 nm	60.5 + /− 7.4
250 nm	56.8 + /− 1.7
350 nm	56.8 + /− 3.8
Ti	69.6 + /− 3.7

### Visualization of biofilms indicates that cell retrieval is similar for both CICNT and Ti surfaces

**Figure 2:**
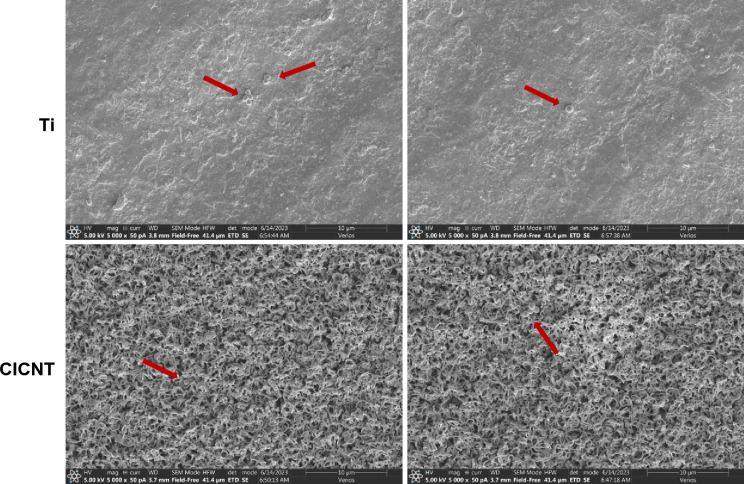
Representative images of bare Ti or CICNT surfaces after performing our protocol for *S. aureus* CFU analysis, indicating that the protocol is successful at removing the biofilm from both Ti and CICNT surfaces. Images were taken using a scanning electron microscope at 5000x. Red arrows indicate remaining bacteria.

We considered the possibility that the differences in CFU counts could be due to a difference in the ability to retrieve cells from Ti vs CICNT surfaces, rather than their ability to adhere. Accordingly, we conducted an experiment where we performed scanning electron microscope (SEM) imaging of both types of surfaces after our typical cell removal process. We found that only very rare cells were still adhered to either CICNT or Ti, suggesting that the cell retrieval process was similar and effective for both surface types (Fig. 2).

### SEM images of mature biofilms on CICNT appear to show fewer cells than biofilms on Ti

**Figure 3:**
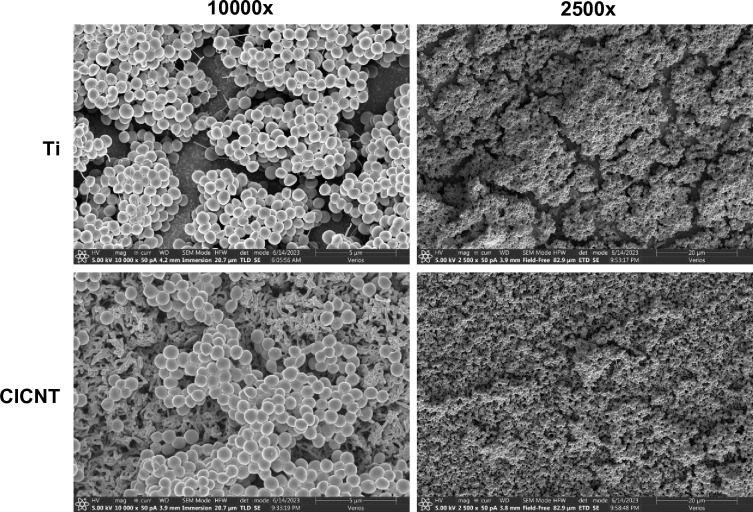
Representative scanning electron microscope images of JE2 biofilms on bare Ti or CICNT surfaces of diameter 150 nm at 10,000× using immersion mode (for greater resolution) and 2500× in a nearby location using field free mode (for a wider view under lower magnification).

In addition to CFU enumeration, we used a scanning electron microscope to image the biofilms grown on both the 150 nm CICNT surface and bare Ti. Since cell counting from an SEM image is inherently less quantitative than CFU enumeration, we performed this experiment as a qualitative companion to the data showing the difference in CFU counts on 150 nm CICNT and Ti. In the SEM images more bacteria can be seen adhered to the Ti surface than to the CICNT surface, and the CICNT surface appears to have more bare spots lacking adherent cells than the Ti (Fig. 3), providing further confirmation of the CFU reduction on the CICNT surface.

### Biofilm growth is slower on CICNT surfaces than on Ti surfaces

**Figure 4:**
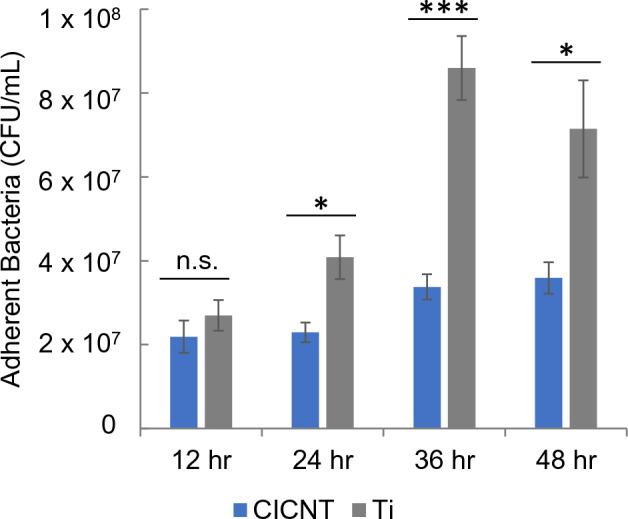
Colony Forming Units/mL + /− standard error quantification of bacteria on CICNT and bare Ti at various time points. Bars represent n = 7 total samples and three independent experiments. *p < 0.01, ***p < 0.0001.

After establishing that the 150 nm CICNT showed the largest reduction in adherent bacteria at 36 h, we worked to better understand this effect over time. To determine if the anti-biofilm effect was present over multiple time points, 150 nm CICNT and Ti control samples were exposed to *S. aureus* for 12, 24, 36, or 48 h and the adherent bacteria were quantified by performing serial dilutions onto agar plates. We also exposed 150 nm CICNT and Ti samples to *S. aureus* for 1 h (data not shown), but because no significant difference was found between adherent bacterial load, we moved to the 12-h time point. At 12 h, no significant difference between the adherent bacterial load was found between the two groups. As time progressed, this difference became significant and the ratio between the number of bacteria on Ti and on CICNT was most substantial at 36 and 48 h (Fig. 4). After determining that the 36-h time point showed the most significant difference in CICNT versus the control, we used that time point for all future experiments. Additionally, we performed a longitudinal analysis and found that the interaction effect of surface type and time was significant. A one-sided general linear hypothesis test on the hypothesis showed that the rate of biofilm growth was lower on the CICNT surface than on the Ti surface was significant (*p* = 0.0014). This indicates that the biofilm was growing more slowly on the CICNT surface than on the Ti surface.

### Differentiating between chemical and structural biofilm resistance

**Figure 5:**
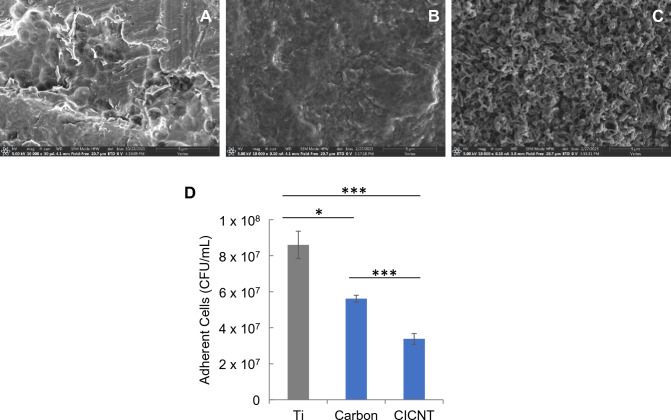
SEM images of (**A**) bare Ti, (**B**) carbon control, and (**C**) CICNT at 10,000×. All images are prior to bacterial growth. (**D**) CFU/mL for bare Ti, carbon control, and CICNT, + /− standard error. Bars represent n = 7 total samples and three independent experiments. *p < 0.05, ***p < 0.0005.

The reduction in adherent bacteria on the CICNT surface could be due to either an anti-adhesion chemical effect of the carbon used, a structural effect of the nanotubes, or both, on a developing biofilm. To differentiate between these two possibilities, we developed a carbon control consisting of nonstructured carbon deposited on the Ti surface. This carbon control, therefore, was chemically similar to the CICNT surface but possessed a different structure. The carbon control was found to have fewer adhered bacteria than the bare Ti surface (*p* = 0.003), but significantly more bacteria than the 150 nm CICNT surface, which is also composed of carbon, (*p* = 4E−5, Fig. 5), indicating that the structure of the carbon affects the number of adhered bacteria.

### A variety of *S. aureus* isolates are inhibited from forming biofilms on CICNT

**Figure 6:**
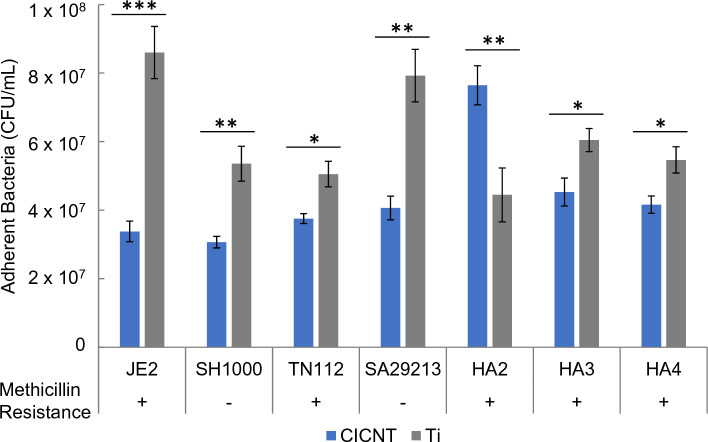
CFU/plate for CICNT and bare Ti with different *S. aureus* isolates grown in biofilms + /− standard error. Bars represent at least n = 7 total samples from at least 3 independent experiments. *p < 0.01, **p < 0.001, ***p < 0.0001.

The previous experiments all used the JE2 strain of *S. aureus*. However, it is also important to understand whether the anti-biofilm effect persists across multiple strains of *S. aureus*. We have previously shown that different *S. aureus* isolates produce biofilms that vary by overall biomass, as well as by polysaccharide, protein and extracellular DNA content^22^. To determine if the reduction in adherent bacteria was consistent among different strains of *S. aureus*, we tested six additional isolates of *S. aureus* (JE2, used in previous experiments, is included as a reference). The clinical isolates chosen were found in a previous publication to represent a variety of relative biofilm strengths, with SH1000 forming the strongest biofilm and HA3 the weakest^22^. Additionally, both methicillin resistant and susceptible isolates were tested. We found that six of the seven isolates exhibited a significant reduction in the number of adherent bacteria on the CICNT surface as compared to bare Ti, while one isolate, HA2, showed a significant increase in the number of adherent bacteria on the CICNT surface (Fig. 6).

## Discussion

The purpose of this study was to investigate the antimicrobial properties of the CICNT surface. CFU analysis demonstrated that the CICNT surface effectively reduces the number of adherent bacteria as compared to bare Ti surfaces, with up to a 2.5-fold reduction in the number of adherent bacteria. SEM analysis confirmed this reduction in adherent bacteria. This reduction is consistent with the lower end of reported bacterial reduction values for the CICNT surface in the only previously published study of the material’s anti-biofilm properties to date^21^, though in that study the CICNT surface modification was prepared on stainless steel rather than Ti. It should also be noted that the methods used by Morco et al. for that original study were different than ours both in their protocol for incubating bacteria on the CICNT surface and in their method for quantifying bacteria after incubation; Morco et al. incubated bacteria on CICNT in a bioreactor in TSB for 24 h, followed by 10% TSB for 24 h, for a total of 48 h and then they counted bacteria remaining on the surfaces using SEM analysis. In this study the bacteria were grown in a still droplet on the CICNT sample surface in 66% TSB, and bacteria were quantified using CFU analysis. It is possible that differences in both biofilm growth conditions and bacterial enumeration played a role in the slight decrease in bacterial reduction found in this study compared to that of Morco et al.

A reduction in adherent bacteria may indicate that a surface is chemically toxic to the cells, causing cell death, commonly referred to as bactericidal. Alternatively, a reduction in adherent bacteria could mean that the structure of the surface is preventing the bacteria from effectively adhering, known as anti-biofouling. SEM images can be used to suggest whether cell death is occurring^10^. SEM images of the *S. aureus* biofilm did not appear to show collapsed or deflated cells on the CICNT surface, suggesting that the reduction in adherent bacteria may be due to a prevention of adhesion rather than cell death. This contrasts with the bactericidal properties of other naturally occurring surfaces with nanoscale topography such as dragonfly and cicada wings^23,24^. An anti-biofouling surface that prevents bacteria from attaching and forming biofilms would play an important role in preventing infection, since antibiotics are much more effective against planktonic cells than against biofilms^25,26^.

The results found in this study indicate that the surface nanostructure of the CICNT is important since different sizes of CICNT exhibit different levels of bacterial reduction. Furthermore, this implies that some material property such as nanotube diameter, curvature, stiffness, or the amount of space between nanotubes impacts the ability of bacteria to adhere to the surface. However, it is unlikely that wettability is a material property that impacts the ability of *S. aureus* to adhere to CICNT surfaces of different sizes since we found no significant difference in water contact angle between surfaces of different diameters. A diameter of 150 nm was found to exhibit the greatest degree of biofilm reduction. This size is within the range of nanostructure sizes found on dragonfly wings, which one study estimated to be between 83.3 and 195 nm^27^, though the topographies of dragonfly wings and CICNT differ. The average size of carbon nanotubes tested in this study ranged from 50 to 350 nm in diameter, which is considerably smaller than that of *S. aureus*, which has a diameter of 0.5–1 μm. The fact that the CICNT surface features are smaller than *S. aureus* is in agreement with previously published literature which demonstrated that bacterial adhesion is reduced on surfaces with nanotopography smaller than the bacteria, possibly due to a reduction in bacteria-surface contact area^28–30^. However, the precise mechanism by which the CICNT surface reduces adherent *S. aureus* bacteria remains to be understood, and is possibly related both to specific mechanical properties of the carbon nanotubes and to the reduction in bacterial surface contact area.

This study also tested biofilm growth at four different time points on both surfaces. At 12 h, there was no significant difference in adherent bacteria between CICNT and Ti. However, at 24, 36, and 48 h there were significantly fewer bacteria on CICNT surfaces than on Ti surfaces. The ratio between the number of bacteria found on Ti and CICNT surfaces was largest at 36 h. The slight decrease at 48 h may be due to our experimental conditions. We also found that the biofilm growth rates differed between surface types. The number of adherent bacteria grew more slowly on CICNT surfaces than on Ti surfaces. This could indicate that either fewer new bacteria were adhering to the surface or that the bacteria present on the surface were not replicating as quickly even though they remained viable. This supports the evidence collected thus far that the CICNT surface possesses anti-biofouling properties.

We also tested the CICNT surface against seven different isolates of *S. aureus*. Most studies of materials suspected to possess anti-biofilm properties are only tested against one or two strains of bacteria^21,31^. However, the composition of the biofilm matrix in *S. aureus* is highly strain-, time-, and condition-dependent^22,32^, so testing multiple strains is important to understand whether a material has broad anti-biofilm capabilities. The three main components of the biofilm matrix are proteins, polysaccharides, and extracellular DNA. These three components are important for adhesion and structural components of the biofilm^33^. The isolates chosen represent both methicillin-resistant and methicillin-sensitive strains, as well as a variety of biofilm matrix compositions^22^. These relative biofilm matrix compositions were determined by Ball et al. by the addition of proteinase K to measure protein, DNase to measure eDNA, or phenol–sulfuric acid to measure polysaccharide^22^. SH1000 was found to have especially robust biofilm formation, while HA3 had relatively weak biofilm formation. HA4 was found to have a biofilm relatively low in eDNA while HA2 and HA3 had relatively high concentrations of eDNA. TN112 and SA29213 had relatively low levels of polysaccharide. HA2, HA3, and HA4 had relatively high levels of protein in their respective biofilm matrices. Beyond matrix composition, methicillin resistance has been shown previously to correspond both to stronger biofilm formation^34^ and in some studies to a more protein- and eDNA-rich biofilm matrix^35^. TN112, HA2, HA3, and HA4 are methicillin resistant while SA29213 and SH1000 are methicillin sensitive. Six of the seven isolates tested showed significantly reduced adhesion on the CICNT surface compared to a bare Ti surface, and the CICNT surface reduced adhesion of both methicillin-resistant and methicillin-sensitive isolates. The HA2 isolate was the only isolate tested which appeared to adhere more strongly to the CICNT surface than to the bare Ti surface. This isolate was found by Ball et al. to have slightly elevated levels of protein and eDNA in its matrix compared to other isolates tested, but was not significantly different from HA3, which was prevented from adhering to the CICNT surface. Given that the other isolates tested showed biofilm reduction on the CICNT surface it is unlikely that either methicillin resistance or biofilm matrix composition was responsible for HA2’s adherence to the CICNT surface. It is possible that some genetic difference between isolates is responsible for the difference in adherence to the CICNT surface, and that future work with additional strains may uncover such a genetic basis for why some isolates are susceptible and some are resistant to the effects of the CICNT surface. Future work will explore both the mechanism behind the response of HA2 to the CICNT surface as well as additional strains and types of bacteria, including gram-negative bacteria, to determine whether the CICNT surface is effective in reducing the number of adherent gram-negative bacteria.

We also compared the growth of bacteria on a non-structured carbon surface to growth on the 150 nm CICNT surface. This unstructured carbon control was found to have significantly more adherent bacteria than the CICNT surface. This indicates that the specific structure of the surface affects the adhesion of the bacteria rather than the carbon itself having an anti-biofilm effect and is in agreement with the results found by Morco et al. with CICNT grown on stainless steel^21^. It also echoes other work which describes how the antimicrobial effect of nanostructured surfaces is heavily affected by their precise arrangement and topographical structure^14,36^. In combination with the results showing that the size of the nanotubes affects how many bacteria attach, this experiment shows that *S. aureus* bacteria are attuned to the precise topographical structure of their environment. Future work will continue to elucidate the mechanisms behind this phenomenon.

## Conclusions

A Carbon-infiltrated carbon nanotube (CICNT) surface modification on Ti surfaces reduced biofilm formation of *S. aureus* and the magnitude of the effect was found to depend upon the CICNT diameter, the *S. aureus* isolate, and the topography of the CICNT surface itself. A CICNT diameter of 150 nm was found to provide optimal protection against adherent *S. aureus*, with a 2.5-fold reduction. This effect was confirmed by CFU quantification and SEM analysis. Multiple isolates of *S. aureus* were investigated, and the antimicrobial effects of the CICNT surface varied by isolate but were shown to inhibit the growth of both methicillin-resistant and methicillin-sensitive isolates. Unstructured carbon did not exhibit the same antimicrobial effect, indicating that the CICNT nanostructure plays a role in biofilm reduction. Further investigation of the mechanism of the antimicrobial effect of the CICNT surface modification is warranted.

## Materials and methods

### CICNT and control sample preparation

**Figure 7:**
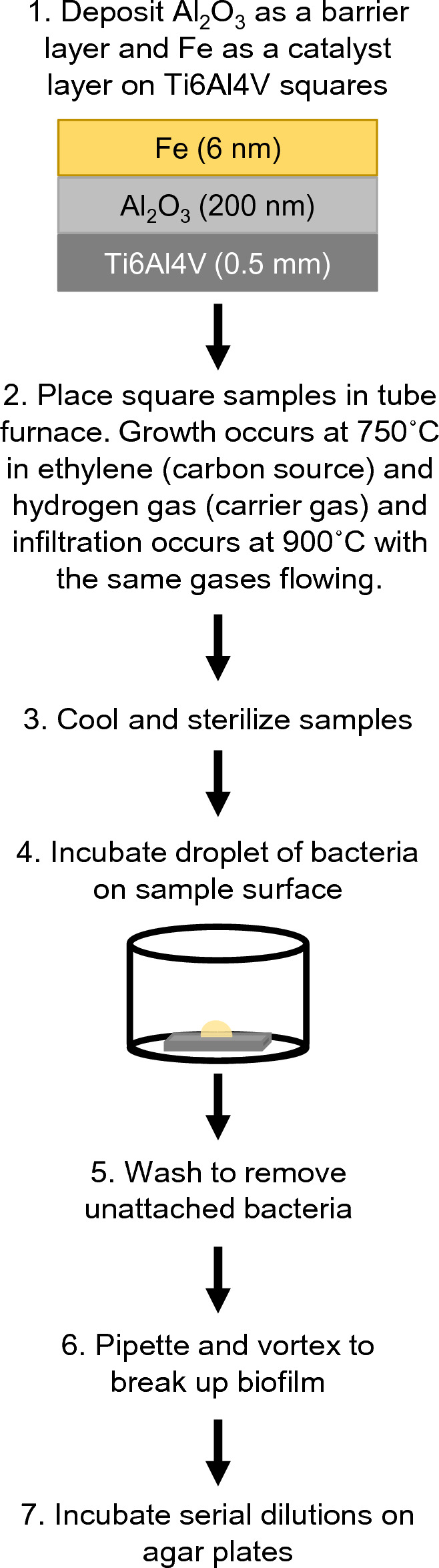
Schematic showing the development and CFU testing of CICNT sample surfaces. Note that the amount of time that the sample surface is exposed to the carbon source ethylene in the furnace controls the size of the resultant carbon nanotubes.

Carbon-infiltrated carbon nanotubes were grown as in previously published literature^21^ with some adjustments (Fig. 7). Briefly, 0.5 mm sheet stock of medical grade Ti6Al4V was cut into 9 mm squares. These squares were sonicated in isopropyl alcohol for 15 min, rinsed in deionized water, and dried. A 200 nm Al_2_O_3_ thin film was deposited on the surface of each square using electron-beam deposition, which was followed by the deposition of 6 nm of iron using a thermal evaporator.

The prepared titanium squares with Al_2_O_3_ and iron thin films were then placed into a furnace for CICNT growth. The furnace was heated to 750 °C with hydrogen gas flowing at 331 standard cubic centimeters per minute (sccm). Once this temperature was reached, ethylene gas flowing at 338 sccm was turned on for a one-minute growth step. The furnace was then heated to 900˚C and a carbon infiltration step was performed with hydrogen and ethylene gas flowing. The infiltration time determined the final average diameter of the nanotubes on a given sample. For 50 nm samples, this step lasted for 2 min, for 150 nm it was 8 min, for 250 nm it was 12 min, and for 350 nm it was 15.5 min. The finished CICNT-coated titanium samples were then cooled in argon flowing at 300 sccm to 200 °C before removal from the furnace.

Carbon control samples were produced by depositing the Al_2_O_3_ layer on Ti6Al4V squares and omitting the iron layer. They were then inserted into the furnace and heated to 900˚C with hydrogen and ethylene gas flowing for 10 min. This procedure produced titanium squares that had been coated with a layer of carbon but did not have nanotube topography (Fig. 5).

All CICNT, carbon control, and titanium control samples were sterilized before exposure to bacteria by the addition of 70% ethanol, followed by three washes with sterile water. Samples were then allowed to dry completely before the addition of bacterial media. This method was tested for sterility, and no bacterial colonies were recovered.

### Bacterial strains

JE2 (BEI Resources NR-46543) is derived from the LAC strain, a well-characterized methicillin-resistant *S. aureus* strain isolated from the Los Angeles County jail in 2002^37^. JE2 differs from the parent LAC strain by the removal of two plasmids^38^. The six other strains were chosen since they represent a variety of biofilm compositions. These strains have previously been tested for their relative composition of biofilm protein, polysaccharide, and extracellular DNA^22^. SH1000 (BEI Resources NR-55396) is a methicillin-sensitive human isolate with the addition of the *rbs*U gene for ribose uptake. TN112 (BEI Resources NR-46261) is a methicillin-resistant USA300 human isolate. SA29213 (ATCC, 29213) is a methicillin-sensitive clinical wound isolate. HA2, HA3, and HA4 are methicillin-resistant clinical isolates donated from a local hospital pathology lab.

### Bacterial culture

Cultures of MRSA SAUSA300_0794 (JE2) were grown up overnight in tryptic soy broth (TSB). They were then diluted to an optical density (OD) of 0.05 in broth consisting of TSB diluted to 66% in sterile water and with 0.5% glucose added^39^. 25 μL of inoculated broth was pipetted as a droplet onto the top of each surface being tested (Fig. 7). The purpose of this droplet method was to prevent confounding results from bacteria growing under the sample surface or on the plastic well. The 25 μL volume was chosen in order to cover the testing surface without running over into the well. This droplet method has been used before with good results^31^. The droplets of bacterial culture were then incubated on the surface of each sample at 37˚C for 36 h unless otherwise indicated. In order to prevent premature evaporation of the droplet, CICNT and Ti samples were exclusively grown in the central wells of a 24-well plate, and the outer wells were filled with sterile water.

### CFU analysis

One of the most common methods for biofilm quantification is crystal violet. However, this method is inappropriate for use with the CICNT surface because the crystal violet dye is trapped by the porous nanotube surface, producing substantial background stain that confounds the results. Therefore, we used serial dilutions and CFU counts for quantification. After bacteria were cultured for the given amount of time, samples were washed once in sterile 1 × phosphate buffered saline (PBS) to remove any unadhered cells and then removed to a well of a new, sterile plate with sterile forceps. 500 μL of PBS was added to the sample surface and pipetted vigorously to dislodge adherent bacteria from the biofilms. The wells containing the PBS, detached bacteria, and appropriate surface were then vortexed for 1 min. 10 μL from each well was then removed and serially diluted in PBS before inoculation on Luria–Bertani (LB) agar plates^22,40^. Plates were then incubated at 37˚C for 24 h. Each test was performed on 7 individual squares of the appropriate surface across three independent experiments. This procedure was confirmed to effectively remove the bacteria by taking SEM images of the samples after the procedure (Fig. 2).

### Water contact angle analysis

To measure the contact angle, a droplet of double distilled water was placed on the surface of each of the following sample types: 50 nm CICNT, 150 nm CICNT, 250 nm CICNT, 350 nm CICNT, and Ti. The water contact angle is the tangential angle to the water droplet at the air–liquid-solid interface^41,42^. Angles were measured using ImageJ and samples were blinded before measurement to eliminate any bias. Reported contact angles are the mean of six total droplets on three individual samples of each material.

### SEM analysis

CICNT and Ti samples with biofilms grown on the surface were prepared for SEM analysis by washing three times with sterile PBS, followed by fixation in 2.5% glutaraldehyde for 2 h. They were then washed with PBS, followed by a wash in sterile water and a dehydration with a graded ethanol treatment for 30 min in 70% ethanol, followed by 30 min in 100% ethanol. Samples were then allowed to dry overnight. A 90-s timed sputter coat of an 80/20 gold/palladium mixture was then applied using a Quorum Q 150 T ES sputter coater. Samples were imaged in a ThermoScientific Verios G4 UC SEM at multiple predetermined locations.

### Statistical analysis

For comparisons of CFU data, significant differences were determined by Student’s *t*-test. Statistically significant differences were attributed to variables with *p* ≤ 0.05. Analysis of data for bacteria grown at different time intervals was performed using a generalized linear model that included a first-order autoregressive correlation structure. An analysis of variance (ANOVA) was performed using this model to determine the significance of bacterial growth rate, and a general linear hypothesis test was performed to compare the CFU counts from different materials in individual time intervals. An ANOVA was also used to determine significance of water contact angles between the five materials tested (50 nm CICNT, 150 nm CICNT, 250 nm CICNT, 350 nm CICNT, and Ti).

### Ethics approval and consent to participate

Approval was granted by the Institutional Biosafety Committee of Brigham Young University to conduct this research (protocol IBC-2018-0046). No human subjects research was conducted in this study.

## Data Availability

All primary data and materials contained in this manuscript are available by contacting the corresponding author (BKB), upon reasonable request.

## Abbreviations

CICNT: Carbon infiltrated carbon nanotubes
PJI: Periprosthetic joint infection
Ti: Titanium
CNT: Carbon nanotubes
SEM: Scanning electron microscopy
CFU: Colony forming unit
PBS: Phosphate buffered saline
LB: Luria–Bertani
OD: Optical density
